# Reactive Oxygen Species and Antioxidative Defense in Chronic Obstructive Pulmonary Disease

**DOI:** 10.3390/antiox10101537

**Published:** 2021-09-28

**Authors:** Akihiko Taniguchi, Mitsuru Tsuge, Nobuaki Miyahara, Hirokazu Tsukahara

**Affiliations:** 1 Department of Hematology, Oncology, Allergy and Respiratory Medicine, Okayama University Academic Field of Medicine, Dentistry, and Pharmaceutical Sciences, Okayama 700-8558, Japan; a-tanig@okayama-u.ac.jp; 2 Department of Pediatrics, Okayama University Academic Field of Medicine, Dentistry, and Pharmaceutical Sciences, Okayama 700-8558, Japan; tsugemitsuru@okayama-u.ac.jp; 3 Department of Medical Technology, Okayama University Academic Field of Health Sciences, Okayama 700-8558, Japan; miyahara@md.okayama-u.ac.jp

**Keywords:** reactive oxygen species, nitric oxide, oxidant, antioxidant, oxidative stress, chronic obstructive pulmonary disease, cigarette smoke, asymmetric dimethylarginine, arginine, biomarker

## Abstract

The respiratory system is continuously exposed to endogenous and exogenous oxidants. Chronic obstructive pulmonary disease (COPD) is characterized by chronic inflammation of the airways, leading to the destruction of lung parenchyma (emphysema) and declining pulmonary function. It is increasingly obvious that reactive oxygen species (ROS) and reactive nitrogen species (RNS) contribute to the progression and amplification of the inflammatory responses related to this disease. First, we described the association between cigarette smoking, the most representative exogenous oxidant, and COPD and then presented the multiple pathophysiological aspects of ROS and antioxidative defense systems in the development and progression of COPD. Second, the relationship between nitric oxide system (endothelial) dysfunction and oxidative stress has been discussed. Third, we have provided data on the use of these biomarkers in the pathogenetic mechanisms involved in COPD and its progression and presented an overview of oxidative stress biomarkers having clinical applications in respiratory medicine, including those in exhaled breath, as per recent observations. Finally, we explained the findings of recent clinical and experimental studies evaluating the efficacy of antioxidative interventions for COPD. Future breakthroughs in antioxidative therapy may provide a promising therapeutic strategy for the prevention and treatment of COPD.

## 1. Introduction

Chronic obstructive pulmonary disease (COPD) is a chronic respiratory disorder characterized by irreversible obstructive breathing. Globally, it is a leading cause of disability and death and exerts a great strain on healthcare resources [1,2]. Various studies on the development of new therapies for COPD and public health approaches are ongoing; however, no radical cure has been found thus far. Furthermore, the complications and quality of life associated with COPD have not been sufficiently improved, and >3 million people die from COPD each year [3,4].

The factors affecting the development and progression of COPD include genetic factors, age, sex, lung growth and development, exposure to particles, socioeconomic status, asthma and airway hyperresponsiveness, chronic bronchitis, and infections [3,5,6]. Among the related particles, cigarette smoke is the most commonly encountered risk factor for COPD [3].

However, the detailed mechanisms by which cigarette smoking causes COPD have not been elucidated. Protease–antiprotease imbalance, neutrophilic airway inflammation induced by immune responses, oxidative stress [7,8,9,10], and apoptosis [11] have been reported to be involved in the development of COPD. However, the fact that only a relatively small percentage of smokers develop COPD [12] and that airway inflammation persists, and the disease progresses even after smoking cessation [13] suggests that other factors, including host genetic factors, are greatly involved in the development and progression of this disease.

COPD is associated with chronic inflammation characterized by an increased number of alveolar macrophages, neutrophils, T lymphocytes (predominantly Tc1, Th1, and Th17 cells), and innate lymphoid cells recruited from the circulation [14,15,16]. These inflammatory cells and structural cells, including epithelial and endothelial cells and fibroblasts, secrete several pro-inflammatory mediators, including cytokines, such as tumor necrosis factor (TNF)-α, interleukin (IL)-1β, IL-6, granulocyte-macrophage colony-stimulating factor (GM-CSF), and IL-8; chemokines, such as C-X-C motif ligand (CXCL) 1; CXCL8, C-C motif ligand (CCL)-2; growth factors, such as transforming growth factor (TGF)-β; and lipid mediators, such as leukotriene (LT) B_4_ [15,17,18,19].

Inflammation in COPD is thought to be closely related to oxidative stress [7,8,9,10,15]. In patients with COPD, oxidative stress occurs because endogenous antioxidant defenses are genetically impaired and/or overwhelmed by the presence of reactive oxygen species (ROS) [7]. One of the sources of ROS is environmental sources, represented by cigarette smoke, and is called exogenous ROS. Conversely, cellular sources, including inflammatory and structural cells, produce ROS upon activation of xanthine oxidase (XO) and nicotine adenine disphosphonucleotide (NADPH) oxidase and/or by mitochondria [7] and are called endogenous ROS. In patients with COPD, oxidative stress occurs after long-term exposure to environmental cigarette smoke and combustion products of biomass fuels, as well as due to a variety of immune and inflammatory stimuli in the airways [20].

Pulmonary inflammation gains strength by increased oxidative stress that subsequently recruits and activates immune cells into the lungs and also produce inflammatory mediators [8]. In this context, a crucial factor that dictates the severity and progression of COPD might be the ability of the host to provide protection against oxidative stress by upregulating lung antioxidant defenses [20,21].

## 2. Cigarette Smoke and Oxidative Stress in COPD

The leading environmental risk factor for COPD is cigarette smoking [3]. Cigarette smoke comprises >4500 components in its gaseous and particulate phases. These compounds include direct carcinogens (methylcholanthrene, benzo-α-pyrenes, and acrolein), toxins (carbon monoxide, nicotine, ammonia, acetone, and hydroquinone), reactive solids with chemically catalytic surfaces [22], and numerous oxidant compounds that have been identified among the 4000–7000 constituents of cigarette smoke [23]. Furthermore, cigarette smoke contains a high concentration of ROS [24] and is considered to be associated with higher levels of oxidative stress in cigarette smokers [25,26]. A single puff of cigarette smoke contains 10^17^ free radicals in the tar phase and 10^15^ free radicals in the gas phase [27,28]. For example, superoxide (O_2_^−^), epoxides, peroxides, nitric oxide (NO), nitrogen dioxide (NO_2_), peroxynitrite (ONOO^−^), and peroxynitrate (O_2_NOO^−^) are present in the gas phase [9].

In addition to inducing ROS, cigarette smoke affects the endogenous oxidant/antioxidant balance and modifies the production of proteins and action of enzymes by epigenetic modulation mechanisms in cells, such as bronchoalveolar epithelial cells and macrophages. Cigarette smoke promotes the activation of inflammatory cells through the activation of transcription factors, such as nuclear factor-κB (NF-κB), p38 mitogen-activated protein kinase (MAPK), and posttranslational modification of histone deacetylase (HDAC) in macrophages [8,29]. These responses trigger the cells to release proinflammatory cytokines, to recruit more neutrophils, macrophages, and dendritic cells and to exacerbate the inflammatory process [8]. Newly recruited inflammatory cells continue the inflammatory process through phagocytosis, cytokine secretion, and surface antigen formation, mediating the innate and adaptive immune responses, while inflammatory and structural cells generate endogenous ROS [8]. The burden of oxidants/ROS in the respiratory tract, through the prooxidant/antioxidant imbalance induced by the activation of inflammatory cells and gene regulatory mechanism described below, results in lung cell damage and induces pulmonary vascular endothelial cell apoptosis [8,30], which delays the resolution of inflammation by compromising the phagocytic ability of alveolar macrophages, leading to necrosis and emphysema [8].

The host’s antioxidant capacity is attenuated by the coordination of cigarette smoking itself, ROS, and various mediators of the associated inflammation, resulting in enhanced oxidative stress [20]. In addition, nitrosative stress is caused by cigarette smoke, which is a source of reactive nitrogen species (RNS). Large amounts of NO, which is abundant in cigarette smoke and is also generated by inducible NO synthase (NOS_2_) in inflammatory cells, reacts with oxygen (O_2_) and O_2_^−^ to produce highly oxidizing NO_2_ and ONOO^−^, respectively [20,31,32].

Furthermore, cigarette smoke is considered to induce apoptosis of pulmonary vascular endothelial cells via epigenetic mechanisms by modulating various regulatory mechanisms, such as deoxyribonucleic acid (DNA) methylation, ribonucleic acid (RNA) methylation, histone modification, microRNA (miRNA), exosomes, and noncoding RNA [30].

Cigarette smoke upregulates mucin 5AC (MUC5AC) expression via activation of activator protein 1 or specificity protein 1 [33,34]. In addition, cigarette smoke constituents, including aldehydes (e.g., acrolein) and hydrogen peroxide (H_2_O_2_), also upregulate mucin gene expression [9]. Cigarette smoke also prevents the apoptosis of airway epithelial cells by suppressing Bik, a proapoptotic molecule, thereby increasing epithelial cell hyperplasia [35].

Long-term exposure to cigarette smoke impairs phagocytosis and antigen-presentation functions of neutrophils and macrophages, which predispose patients to respiratory tract infections. Chronic inflammation and bacterial colonization in the lower respiratory tract, combined with compromised host defenses, could explain the progression of COPD even after smoking cessation [8].

## 3. Other Sources of Oxidative Stress in COPD

It has been reported that approximately 25–45% of patients with COPD have never smoked; therefore, we cannot neglect other risk factors of COPD that can induce oxidative stress leading to COPD [36]. Exposure to biomass has a 2.5–3 times higher risk of airflow limitation; especially, the use of biomass fuel for cooking in low-and-middle income countries is assumed to be one of the important risk factors for COPD [37]. Occupational exposure to dust, gas, and fumes was also significantly associated with an increased risk of COPD [38,39].

The association between ambient air pollution and the incidence of COPD has also been reported. Ozone induces bronchial inflammation through oxidative injury. It has been reported that ozone level is associated with COPD incidence [40]. Associations of other ambient air pollutants, such as particulate matter 2.5 and NO_2_, with the incidence of COPD have also been reported [41,42].

## 4. Oxidative Stress in the Development and Progression of COPD

Enhancement of oxidative stress is thought to be caused by the coordination of exogenous oxidants, such as cigarette smoke, biomass smoke, and air pollution; endogenous ROS, such as O_2_^−^ and H_2_O_2_; and reduction of antioxidants, such as superoxide dismutase (SOD), glutathione (GSH), and nuclear factor erythroid 2-related factor 2 (Nrf2) [20]. Enhanced oxidative stress triggers various reactions, including enhancing inflammation through the activation of transcription factors (such as NF-κB and p38 MAPK), producing autoantibodies to activate autoimmunity and lowering antiproteases to promote emphysema [20].

Macrophages play a key role in orchestrating chronic inflammation in patients with COPD and can be activated by cigarette smoke extract to release inflammatory mediators, including TNF-α, CXCL1, CXCL8, CCL2, LTB_4_, and ROS [15]. Under the direction of these chemotactic mediators, neutrophils migrate into the respiratory tract and secrete serine proteases, including neutrophil elastase, cathepsin G, and proteinase-3, which damage tissues and stimulate mucus secretion from submucosal glands and goblet cells. Neutrophils also secrete matrix metalloproteinase (MMP)-8 and MMP-9, which contribute to alveolar destruction and development of emphysema [15,43,44].

Decreased antioxidants also contribute significantly to increased oxidative stress. Antioxidants, such as SOD, GSH, thioredoxin, and Nrf2, are considered to play very important roles in the lungs, which are continuously exposed to the external environment. Expression of extracellular SOD3 polymorphisms is reduced around small airways in patients with COPD [45], and the transcription factors that regulate multiple antioxidant genes, Nrf2 and FOXO3a (Forkhead box O3a), are reduced in lungs with COPD [46,47]. Nrf2 is a transcription factor that is involved in protection against oxidative damage by regulating the expression of genes, and its endogenous inhibitor, Kelch-like ECH-associated protein 1, prevents binding to the antioxidant response element [48]. Moreover, disruption of Nrf2 causes early onset and severe emphysema, and Nrf2 activator, 1-[2-cyano-3-,12-dioxooleana-1,9(11)-dien-28-oyl] imidazole, attenuates cigarette smoke-induced emphysema and alveolar/cardiac dysfunction in mice [49]. Even after cessation of particle exposure, COPD exacerbation continues by reducing the host antioxidant capacity, and oxidative stress is sustained after amelioration of exacerbations due to continuous production of ROS from endogenous sources, similar to the continuation of oxidative stress after smoking cessation [7].

Over 50 cytokines and chemokines are released in the lungs of patients with COPD and contribute to increased inflammation [50]. Many of the intracellular signaling pathways that trigger and/or drive the release of these inflammatory mediators are sensitive to oxidative stress due to the incorporation of redox-sensitive molecular targets, such as NF-κB, Ras/Rac, Jun-N-terminal kinase, p38 MAPK, and protein tyrosine phosphatases [20]. Oxidative stress activates NF-κB and TGF-β signaling pathways in airway epithelial cells and macrophages, which induce oxidative stress [20] and are involved in small airway fibrosis. As a result of the activation of these intracellular signaling pathways triggered and regulated by oxidative stress, the airway lumen of patients with COPD is characterized by an increased number of neutrophils, macrophages, T lymphocytes, and B lymphocytes. This inflammation is more amplified in patients with COPD than in smokers without COPD and increases further during acute exacerbations or when precipitated by bacterial/viral infection [15].

Oxidative stress also regulates the expression of mucin genes, such as MUC5AC and mucous cell metaplasia [9]. Exposure to H_2_O_2_, which is a constituent of cigarette smoke and an endogenous oxidant, upregulates MUC5AC mRNA expression via an NADPH oxidoreductase 4-dependent mechanism [51].

**Figure 1:**
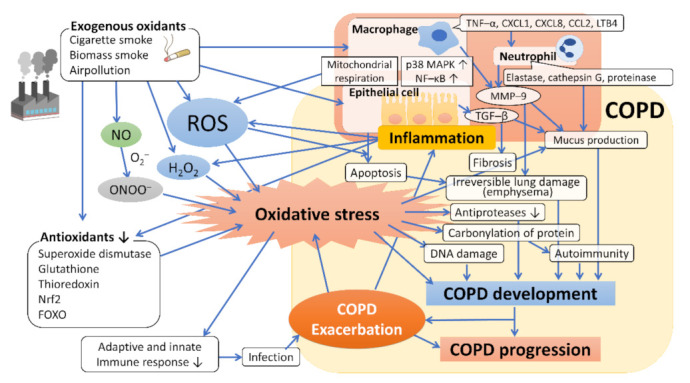
Oxidative stress and the mechanism underlying the development and progression of COPD. Exogenous oxidants such as cigarette smoke contain ROS, which act to promote oxidative stress and induce inflammation of macrophages and bronchoalveolar epithelial cells by activating transcription factors such as NF–κB, which leads to further ROS generation (endogenous ROS). The host’s antioxidants, such as superoxide dismutase, glutathione, thioredoxin, and Nrf2, are attenuated by the coordination of exogenous oxidants and various mediators of the associated inflammation, resulting in enhanced oxidative stress. Oxidative stress regulates the expression of the mucin gene to increase mucin secretion, inducing DNA damage and causing autoimmunity through carbonylation of proteins, and reduces antiprotease activity, leading to the development and progression of emphysema and fibrosis. Abbreviations: ROS, reactive oxygen species; H_2_O_2_, hydrogen peroxide; NO, nitric oxide; O_2_^–^, superoxide anion; ONOO^–^, peroxynitrite; NF–κB, nuclear factor–κB; p38 MAPK, p38 mitogenactivated protein kinase; Nrf2, nuclear factor erythroid 2–related factor 2; FOXO, forkhead box O; TNF–α, tumor necrosis factor–α; LTB4, leukotriene B4; GM–CSF, granulocyte macrophage colony–stimulating factor; TGF–β, transforming growth factor–β; MMP–9, metalloproteinase–9.

In severe COPD, autoantibodies against epithelial and endothelial cells are generated, causing autoimmunity. Oxidative stress is considered to cause carbonylation of proteins, which creates neoantigens and induces the production of autoantibodies [20]. Furthermore, oxidative stress directly damages the DNA. Apurinic/apyradymic sites, which are common DNA lesions in the repair of oxidized bases, are increased in the lungs of smokers without COPD, reflecting active DNA repair, whereas they are reduced in the lungs of patients with COPD, indicating defective DNA repair [20]. Many of these responses to oxidative stress may eventually be involved in the development and progression of COPD; moreover, acute exacerbation causes a further increase in oxidative stress, which also leads to the progression of COPD. A summary of these points is presented in Figure 1.

## 5. Oxidative Stress and Vascular Endothelial Dysfunction

NO is synthesized by NO synthases (NOSs) in every cell type from L-arginine and molecular oxygen; simultaneously, citrulline is formed. NOSs exist in three isoforms: neuronal (NOS1), inducible (NOS2), and endothelial (NOS3) and differ in the way their activity is controlled. NOS1 and NOS3 are fundamentally present in various cells and are activated by a transient increase in intracellular calcium concentrations. NOS2 is induced in response to inflammatory and immunological stimuli in many cell types, including smooth muscle cells, vascular endothelial cells, hepatocytes, glial cells, and activated immune cells [31,52,53].

The vascular endothelium is a widely distributed organ responsible for the regulation of blood flow; control of vascular permeability; angiogenic vascular remodeling; and metabolic, synthetic, anti-inflammatory, and anti-thrombogenic processes, apart from being a mere barrier between intravascular and interstitial compartments. Understanding the relationship between NO system blockade, endothelial dysfunction, and oxidative stress is expected to enable the description of a rational therapeutic strategy in conditions associated with oxidative stress enhancement [31,53,54]. NO from the vascular endothelium is also an antioxidative factor that neutralizes O_2_^−^. Increased oxidative stress causes vascular endothelial dysfunction, whereas NO deficiency increases oxidative stress [55,56].

Asymmetric dimethylarginine (ADMA), which inhibits NOS, is also formed endogenously. During protein synthesis, the arginine residue in the posttranslational peptide is methylated by protein arginine methyltransferase (PRMT). This residue is cleaved from the protein and released from the cell by ADMA. ADMA is degraded into citrulline and dimethylamine by the action of dimethylaminohydrolase (DDAH). DDAH is distributed in tissues and cells in a manner almost identical to that of NOS [57]. When ADMA is administered to healthy persons, blood vessels become constricted, and blood pressure is elevated. In addition, because PRMT activity increases and DDAH activity decreases owing to increased oxidative stress, the ratio of ADMA to NO from the vascular endothelium increases, and NO production decreases further [46,48]. ADMA is not only a factor that facilitates vascular damage but is also a biomarker that is useful in estimating the prognosis of cardiovascular and kidney diseases. A reliable enzyme-linked immunosorbent assay (ELISA) for ADMA was developed by Schulze et al. [58,59]. Dysregulation of the balance between the L-arginine–NOS–NO and PRMT–ADMA–DDAH pathways is thought to be involved in the accumulation of ADMA in tissues and blood, thereby contributing to cardiovascular pathologies.

Recent evidence has indicated that ADMA plays an important role in lung disease, more specifically in COPD. Furthermore, cigarette smoke-induced endothelial dysfunction/injury is linked to pulmonary lesions in COPD (especially emphysema) and systemic comorbidities, including atherosclerosis, pulmonary hypertension, and chronic kidney disease [60]. Several studies have indicated that patients with COPD have significantly higher circulating concentrations of ADMA than healthy controls [61]. Aydin et al. also reported a significant association between ADMA concentrations and progression of COPD and significant inverse correlations between ADMA concentrations and forced expiratory volume in 1 s (FEV_1_) and between ADMA and NO in patients with COPD [62]. In a prospective study of 150 patients admitted to the hospital with acute exacerbation of COPD, Vögeli et al. reported that circulating ADMA concentrations were not independently associated with all-cause mortality at 30 days and 1 year; however, ADMA concentrations independently predicted all-cause mortality at 6 years [63]. Further interventional trials are needed to evaluate whether therapeutic modification of the L-arginine–NO and ADMA pathways has the potential to improve outcomes in patients with COPD.

## 6. Citrulline–Arginine and Arginine–Ornithine Pathways

The arginine metabolic system is important in organ associations. The arginine–NO–citrulline (NO formation) and arginine–ADMA–citrulline (ADMA formation) systems are linked to citrulline–arginine recycling. Citrulline–arginine recycling mainly occurs in blood vessels and the kidneys. Arginine is regenerated from citrulline. The vascular endothelium maintains NO synthesis via this pathway. Increased oxidative stress decreases recycling in the vascular endothelium and decreases NO levels in the endothelium [31,64].

**Figure 2:**
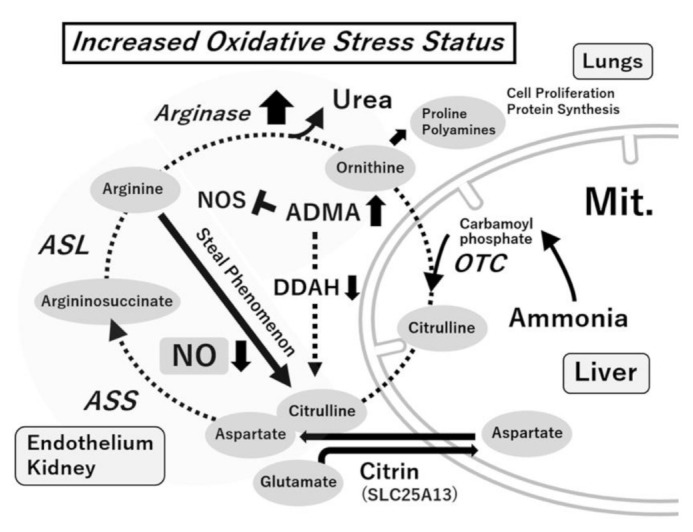
Metabolic network centered on arginine. In the physiological state, arginine is regenerated from citrulline by citrulline–arginine recycling in the blood vessel and the kidney, and the vascular endothelium cells can maintain NO synthesis via this pathway. In inflammatory pathology (increased oxidative stress status), arginase is induced in many cells, such as the blood vessels and macrophages, and NO synthesis derived from the vascular endothelium decreases through the “arginine steal phenomenon”. Increased catabolism of arginine via arginase may not only compromise the ability to synthesize NO constitutively but also increase the production of ornithine, a precursor for the synthesis of proline and polyamines, which are required for cell proliferation and collagen synthesis in the lungs. Abbreviations: ADMA, asymmetric dimethylarginine; ASL, argininosuccinate lyase; ASS, argininosuccinate synthetase; DDAH, dimethylaminohydrolase; NO, nitric oxide; NOS, nitric oxide synthase; OTC, ornithine transcarbamylase; PRMT, protein arginine methyltransferase.

Arginase, the last enzyme in the urea cycle, hydrolyzes arginine to form polyamines and proline. Arginase I is present mainly in the liver, whereas arginase II is present mainly in the mitochondria of cells outside the liver. NOS and arginase, which share arginine as a substrate, control each other’s functions. Arginase I is induced in many cells, including vascular endothelial cells, smooth muscle cells, hepatocytes, and macrophages in inflammatory pathology (“arginine steal phenomenon”) (Figure 2) [65]. When the activity of both NOS and arginase I increases, NOS uncouples are caused by decreased arginine, resulting in decreased NO formation and increased O_2_^−^ formation. Blood arginase I levels are high in patients with bronchial asthma or similar diseases. The effects of arginase I inhibitors (such as N^ω^-hydroxy-nor-l-argininenor-NOHA and S-(2-boronoethyl)-L-cysteine) have also been observed in animal models of airway inflammation [66,67].

**Figure 3:**
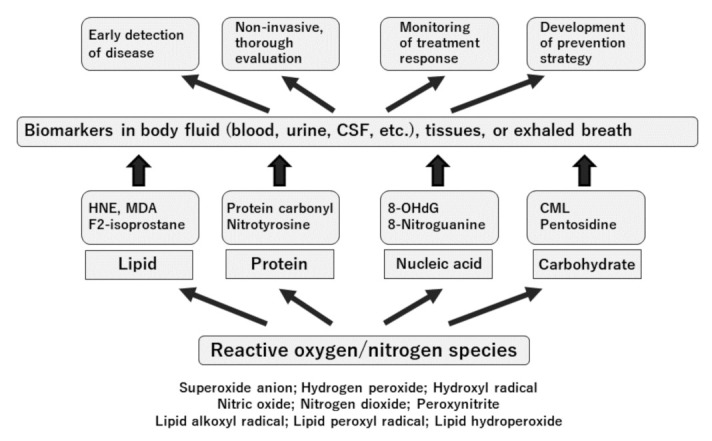
Perspective on the clinical use of oxidative stress biomarkers. Abbreviations: CML, carboxymethyl lysine; CSF, cerebrospinal fluid; HNE, 4-hydroxy-2-nonenal; MDA, malondialdehyde; 8-OHdG, 8-hydroxy-2′-deoxyguanosine. Only the representative markers are presented here.

The two pathways of NO and ADMA formation, as well as citrulline–arginine recycling, are also involved in the urea cycle. This is related to the organ network centered on blood vessels and the kidneys. From the viewpoint of biomarkers, the ornithine–arginine ratio in the blood is likely to be an indicator of the arginase activity, the citrulline + ornithine–arginine ratio is an indicator of the NOS and arginase activity, and the arginine–ADMA ratio is an indicator of the NOS substrate–NOS inhibitor ratio (Figure 3) [64].

In patients with COPD, the levels of oxidative stress biomarkers, carbonyls, and malondialdehyde increase. Augmented platelet and erythrocyte arginase activities associated with cigarette or wood smoke exposure have also been reported [68]. In the airways, NOS and arginase I compete for the common substrate L-arginine. In chronic airway diseases, such as asthma and COPD, elevated arginase expression contributes to airway contractility, hyperresponsiveness, inflammation, and remodeling [69]. Moreover, arginase inhibition has been shown to protect against the development of COPD-like inflammation and remodeling in a guinea pig model of COPD [70]. In an animal model, arginase inhibition shifted the ornithine–citrulline ratio toward citrulline and prevented neutrophilia, mucus hypersecretion, airway fibrosis, and pulmonary hypertension. Increasing the availability of substrate for NOS by arginase inhibition or supplementation with L-arginine, L-citrulline or a combination thereof can also be applied to human COPD [71].

## 7. Oxidative Stress and Oxidative Stress Biomarkers

The expression of cellular physiological function requires the tonic formation of ROS, which is indispensable for functions, such as signaling, organelle function, energy production, and processing of unnecessary cells and foreign matter [32,72].

Oxidative stress is defined as an imbalance between prooxidants and antioxidants in favor of the former, leading to the disruption of redox signaling and control. When ROS is formed excessively in the body, they damage the lipids, proteins, and enzymes responsible for the biological structure and function and genomic DNA responsible for genetic information, thereby damaging tissues and organs and causing disease. Consequently, a vicious cycle ensues in which oxidative stress is amplified, causing disease progression and damage to other organs. Although the body addresses increased oxidative stress using the organ network, tissue damage advances when stress is severe, prolonged, or incessant [31,73]. The formation and elimination of ROS are precisely controlled by the antioxidative system. “Increased oxidative stress” or “redox control failure” is involved in the progress of many kinds of human diseases [74].

Oxidative stress levels in the body can be accurately evaluated by measuring the biological components that are oxidatively modified or damaged [75]. The requirements for a biomarker of oxidative stress include a biomarker that is only slightly accumulated, is not metabolized, and is stable in the body. The emerging availability of ELISA for oxidative stress status markers allows its application to the assessment of various pathological conditions [76,77]. Recently, instruments that can rapidly measure blood hydroperoxides, biological antioxidative potential, urinary 8-hydroxy-2-deoxyguanosine (8-OHdG), and L-type fatty acid binding protein from a minute amount of sample (approximately in 10 min) have been developed, and the oxidative stress environment can be evaluated at bedside [78].

Furthermore, evaluating oxidative stress markers in exhaled breath has been proved to be useful in managing various airway inflammatory diseases [79,80,81]. These “lung biomarkers” may be helpful in diagnoses, defining specific phenotypes of diseases, monitoring exacerbations, and assessing the effects of therapeutic agents in airway diseases.

The important advantages of biomarker measurement in clinical practice are as follows: (1) the therapeutic effects can be evaluated noninvasively and minimally invasively (for patient-friendly continuous evaluation), (2) the pathology can be analyzed from the viewpoint of biological response (for rational multidisciplinary treatment), (3) similar markers can be measured in model animals (for optimal in translational research), and (4) rapid tests are applicable (for proper judgment at an early stage).

The clinical application of “antioxidants” in diseases has the following requirements: (1) the drug shows antioxidative properties at the level of cells, tissues, or organs; (2) oxidative stress is involved in the onset or deterioration of the disease; (3) the drug is effective in a disease model; (4) there is a history of use of the drug in humans; and (5) clinical studies in humans have been performed. The well-known drugs that meet these five requirements and are used in clinical situations include edaravone in the brain field, hydroxymethylglutaryl-CoA reductase inhibitors in the cardiovascular field, and angiotensin-converting enzyme and angiotensin 1 receptor inhibitors in the kidney. Oxidative stress marker measurement is translational in (1)–(5). Figure 3 presents a summary of the significance of oxidative stress markers in clinical medicine.

## 8. Evaluation of Oxidative Stress Status in the Blood of Patients with COPD

Oxidative decomposition of lipids by ROS, called lipid peroxidation, results in the formation of highly reactive and unstable lipid peroxides. Malondialdehyde (MDA), 4-hydroxyhexenal, and thiobarbituric acid-reactive substances (TBARS) are by-products formed by the decomposition of lipid peroxide [82]. In previous studies, plasma TBARS and MDA levels in patients with COPD were higher than those in healthy controls [83,84]. Furthermore, the levels of plasma MDA in healthy controls were increased in parallel with the severity of COPD and also significantly increased during acute exacerbations of COPD [85,86]. Moreover, other biomarkers associated with lipid peroxidation in the plasma (lipid peroxide, conjugated diene, oxidized low-density lipoprotein, and 8-isoprostane) were significantly higher in patients with COPD than in healthy controls [87,88,89].

Protein carbonylation is an ROS-induced protein modification that mediates redox signaling. Previous studies have reported a significant increase in protein carbonyl groups in the plasma samples from patients with COPD compared with samples from healthy controls and its correlation with disease progression [90,91]. In another previous study, advanced oxidation protein products were increased in the plasma of patients with COPD compared with that of healthy controls [92].

Oxidative stress is quantified rapidly, including the measurement of total hydroperoxides (TPs), which function as a marker of overall oxidative injury. TPs are an intermediate oxidative product of lipids, peptides, and amino acids. In addition, the biological antioxidative potential (BAP) is used to assess the total antioxidative activity. TP and BAP measurement is a simple and informative outpatient or bedside measurement in clinical medicine [75]. An increase in TPs was observed in patients with COPD compared with controls [93]. A previous study reported a significant reduction in the BAP in patients with COPD compared with healthy controls and a significant decrease in the BAP during COPD exacerbation [90].

8-OHdG is a DNA structure in which the 8-position of deoxyguanosine is hydroxylated. 8-OHdG is one of the most widely used oxidative stress markers [76,94]. The levels of 8-OHdG were significantly higher in the blood of patients with COPD than in the blood of healthy controls [91].

Thiols are organic compounds that contain sulfhydryl groups (–SH). Thiols in the plasma mainly comprise protein thiols and partially comprise low-molecular-weight thiols, such as cysteine, cystenylglycine, GSH, homocysteine, and γ-glutamylcysteine, which are antioxidants in the plasma [95]. A previous study indicated a significant reduction in protein SH groups in the blood of patients with COPD compared with that of healthy controls and a significant reduction in the protein SH group in COPD exacerbation [96]. GSH normally exists as reduced GSH in the body; however, as reduced GSH is converted to oxidized glutathione (GSSG) during oxidative stress, the GSH–GSSG ratio is also used as an index of oxidative stress status. Compared with healthy controls, patients with COPD were found to have lower plasma GSH levels [97]. No significant difference was found in the amount of reduced GSH, depending on the severity of COPD [98]. The total GSH–GSSG ratio was shown to decrease during COPD exacerbation [99].

Antioxidant enzymes are important components of the antioxidant defense system. SOD is a metalloprotein that removes O_2_^−^ and converts it to H_2_O_2_ [100]. As per previous studies, an increase or decrease in SOD activity was observed in the blood of patients with COPD compared with that in healthy controls [101,102]. No significant difference in SOD activity based on the severity of COPD has been reported [103]. In addition, SOD activity increases during COPD exacerbation [104]. Catalase catalyzes the breakdown of H_2_O_2_ into water and oxygen [105]. Decreased blood catalase activity was observed in patients with COPD compared with that in healthy controls [86]. In another study, catalase activity was significantly decreased in patients with moderate-to-severe COPD [85]. GSH peroxidase promotes the reaction between H_2_O_2_ and reduced GSH to produce H_2_O and GSSG [106]. It was further reported that glutathione peroxidase (GSH-Px) activity in the blood was decreased in patients with COPD compared with healthy controls, and plasma GSH-Px activity was decreased in parallel with the severity of COPD [90,103]. Glutathione-S-transferase (GST) is an enzyme that binds to GSH to detoxify and to neutralize its activity [107]. Plasma GST activity decreases in patients with COPD [108].

Paraoxonase 1 (PON1) is an enzyme present in the blood that binds to apolipoprotein A1 of high-density lipoprotein and exhibits oxidative protection of lipoprotein [109]. There was a significant difference in PON1 activity between patients with COPD and healthy controls [110]. A summary of this section is provided in Table 1.

## 9. Oxidative Stress Biomarkers in Exhaled Breath of Patients with COPD

As described in the preceding section, the assessment of airway oxidative stress using “lung biomarkers” has become an important tool in the early diagnosis of preclinical stage conditions, prevention of exacerbations, and accurate monitoring of management outcomes [79,81].

The concentration of fractional exhaled NO (FeNO) is useful in assessing airway inflammation and is being increasingly used as a biomarker for eosinophilic airway inflammation and response to corticosteroid therapy [81]. However, in patients with COPD, FeNO does not necessarily reflect airway inflammation or disease severity. Evaluation of oxidative stress markers using exhaled breath condensate is also a noninvasive inspection method that reflects the degree and severity of airway inflammation in COPD [129].

Most of the exhaled breath components are water vapor, although a small amount of aerosolized airway coating liquid components is also present. Therefore, it is possible to directly measure various types of mediators in airway mucosal fluids and, thus, the oxidative stress status in the lungs or bronchi. Because the exhaled breath condensate can be collected under normal resting breathing, this test can be performed repeatedly in a simple and noninvasive manner. In addition, several portable collection devices are commercially available, making it easier to perform at the bedside.

To date, several oxidative stress markers in exhaled breath condensates have been reported to be increased in patients with COPD. The concentration of H_2_O_2_ was increased in the exhaled breath condensate of patients with COPD during the exacerbation of COPD [130]. H_2_O_2_ was increased further in exhaled breath condensate in moderate and severe COPD compared with that in mild COPD [131]. In addition, increased H_2_O_2_ in exhaled breath was associated with a decrease in FEV_1_, an increase in neutrophil count, and an increase in the dyspnea score [131,132].

8-Isoprostane is produced by the peroxidation of arachidonic acid and can be a useful marker of oxidative stress in the lungs, measured using exhaled breath condensate [133]. 8-Isoprostane concentration in exhaled breath condensate was increased in patients with COPD, and it correlated with the severity of COPD [131,134]. The levels of 8-isoprostane in patients with stable COPD were higher than those in healthy controls and lower than those in patients with COPD exacerbation [135]. In addition, a correlation between the levels of 8-isoprostane and prevalence of emphysema on high-resolution computed tomography has been reported [136]. However, there was no correlation between the increase in 8-isoprostane in exhaled breath condensate and predicted FEV_1_/forced vital capacity ratio, and a decrease in inhaled steroid treatment was not observed. No correlations were observed between 8-isoprostane and smoking history, sputum cells, or the dyspnea scores in patients with COPD [137]. MDA levels in exhaled breath condensate were reported to be increased in patients with stable COPD compared with those in control smokers or non-smokers [138].

NO formation by NOS2 is increased under oxidative conditions, such as cigarette smoking, and NO reacts with ROS to form nitrite (NO_2_^−^), nitrate (NO_3_^−^), and ONOO^−^ [139]. Nitric oxide-related compounds, such as NO_2_^−^, NO_3_^−^, and S-nitrosothiols [140], can be measured using exhaled breath condensate. In COPD, the concentrations of NO_2_^−^, ONOO^−^, and 3-nitrotyrosine [141] were increased. Higher levels of NO_2_^−^ were observed in patients with COPD than in smokers or nonsmokers, and NO_3_^−^ levels in COPD showed a similar profile [142]. There was an increase in S-nitrosothiols in the exhaled breath condensate of patients with COPD, although there was no apparent difference compared with that in normal smokers.

## 10. Oxidative Stress and Genetic Factors

Advances in genetic approaches have successively revealed the genes associated with pulmonary dysfunction and emphysema in COPD [143], several of which are related to the oxidative stress status [144]. Using a candidate gene approach, several genetic mutations in antioxidant genes have been linked to COPD severity [9]. In a case–control study of 346 subjects with and without COPD, Vibhuti et al. examined the polymorphisms of 462Ile/Val, 3801T/C of *CYP1A1*, −3860G/A of *CYP1A2*, and −930A/G, 242C/T of *CYBA* individually and reported that *CYP1A1*, *CYP1A2*, and *CYBA* gene polymorphisms are associated with oxidative stress in COPD [112]. According to a review by Postma et al. [144], polymorphisms in *GSTM1*, *GSTP1*, *SOD3*, and *EPHX1* genes are associated with a more rapid decline in lung function in COPD.

Malhotra et al. reported that HDAC2 downregulation impairs Nrf2 activation in the lungs by decreasing the half-life of Nrf2 [145]. Sirtuin (SIRT) 1 and SIRT6, class III HDACs that catalyze nicotinamide adenine dinucleotide (NAD)^+^-dependent deacetylation, are downregulated in the peripheral lungs of patients with COPD [146,147], and loss of SIRT1 correlates with tissue inhibitor of metalloproteinase (TIMP)-1 lysine acetylation and subsequent degradation of TIMP-1, the major anti-MMP protease, resulting in increased MMP-9 in human COPD lung tissue [148]. Through in vitro studies, Baker et al. showed that H_2_O_2_ selectively elevates miR-34a, leading to reduction of SIRT1/6 in bronchial epithelial cells, while inhibition of miR-34a (antagomir) increased SIRT1/6 [149].

Recently, genome-wide association studies have reproducibly identified genes associated with lung function and COPD. Among them, the GST C-terminal containing domain has one of the genes most closely related to oxidative stress. It is located on chromosome 4q24 and is associated with detoxification of products of oxidative stress and synthesis of steroid hormones [150]. Other genes include *AGER* and *FAM13A* [143]. *AGER* is a gene encoding Receptor for advanced glycation end-product (RAGE), and one of the ligands for RAGE is advanced glycosylation end-product (AGE), whose secretion is promoted by oxidative stress. The expression of RAGE and level of its ligands are enhanced in the lungs, mediating the inflammatory response in COPD [17,151,152]. Cigarette smoke extract induces the alteration of RAGE distribution via the activation of redox-sensitive damage-associated molecular pattern (DAMP) signaling through Nrf2 in cells, whereas the inhibition of RAGE exhibits anti-inflammatory and antioxidative/nitrosative effects through the inhibition of Nrf2/DAMP signaling [153].

*FAM13A* expression is associated with the risk of COPD. In mitochondria, it regulates the expression of carnitine palmitoyl transferase-1A, which is upregulated by cigarette smoke and fatty acid oxidation via the intermediate protein SIRT1 [154]. It has also been reported that the deficiency of alpha-1-antitrypsin (AAT) translated from *SERPINA1*, a major genetic risk for COPD, contributes to oxidative stress [155]. The results are summarized in Table 2.

## 11. Treatment Strategy for COPD Focusing on Oxidative Stress

As discussed above, oxidative stress is a major driving force underlying the pathophysiology of COPD [10]. Therefore, reducing oxidative stress is an important therapeutic strategy [20].

Administration of antioxidants and enhanced expression of endogenous antioxidants may reduce oxidative stress in COPD and may therefore be useful for treatment. The thiol compound, developed as a mucolytic agent, is also known as an antioxidant. Clinical trials, such as those investigating N-acetylcysteine (NAC), carbocysteine, and erdosteine, have been conducted, and exacerbation-suppressing effects have been reported. NAC donates cysteine intracellularly to promote GSH production and has an antioxidant effect [160]. In the Bronchitis Randomized on NAC Cost-Utility Study, low-dose NAC was administered to patients with COPD, and disease exacerbation was suppressed in patients who were not treated with inhaled steroids [161]. In the PANTHEON study in China, high-dose NAC administration was administered to patients with COPD, with a reduction in acute exacerbation of COPD observed regardless of inhaled corticosteroid use [162].

Carbocysteine is a thiol mucolytic agent with antioxidant properties. In a randomized placebo-controlled study conducted in Japan and China, carbocysteine was administered to patients with moderate-to-severe COPD to suppress the exacerbation of COPD [163]. These are relatively well-tolerated agents and may be beneficial for the clinical management of COPD. However, it is unclear whether the suppressive effect of COPD exacerbation is due to the improvement of mucus clearance by mucus decomposition or antioxidant effect in the lungs.

The most encouraging approaches to antioxidant therapy are the use of Nrf2 activators, which activate multiple antioxidant genes and address the defect in Nrf2 response to oxidative stress that occurs in patients with COPD [164]. Nrf2 levels are usually reduced in alveolar macrophages derived from patients with COPD [165]. As COPD progresses, the expression of Nrf2 in the lungs decreases. An increase in the incidence of emphysema due to cigarette smoke exposure has been observed in Nrf2-deficient mice [166]. Nrf2 activators may contribute to reducing oxidative stress in the lungs of patients with COPD and act protectively against ROS in cigarette smoke.

Nrf2 activators, such as sulforaphane, bardoxolone methyl, and dimethyl fumarate, are currently being investigated as therapeutic agents. Sulforaphane is a compound found in various vegetables, including broccoli, radish, cabbage, cauliflower, and wasabi, which promote the expression of multiple Nrf2-mediated antioxidant genes [167]. It also improves the corticosteroid susceptibility in patients with COPD. However, in clinical trials of sulforaphane in patients with COPD, an increase in antioxidant gene expression was not observed, and oxidative stress could not be suppressed [168]. Synthetic triterpenoid (bardoxolone methyl) is also an Nrf2 activator that has been shown to suppress the development of emphysema in cigarette smoke-exposed mouse models [49]. Dimethyl fumarate is also an Nrf2 activator, although its use is associated with side effects, such as flushing, nausea, and diarrhea [169]. It is crucial to develop potent and specific Nrf2 activators; therefore, it is an important area for drug development in the future.

Dietary antioxidant supplementation may improve and support the antioxidant defense mechanisms in the body. There is insufficient evidence supporting the improvement in lung function and clinical manifestations of COPD with the use of antioxidant vitamins (such as vitamin C and vitamin E) and food antioxidants (such as polyphenols, including resveratrol, quercetin, and curcumin). Vitamin C (ascorbic acid) and vitamin E (α-tocopherol) were investigated for clinical application in COPD, with the expectation of antioxidant effects. A positive correlation was found between vitamin E intake and lung function in patients with COPD, suggesting that vitamin antioxidant intake regulates COPD-associated oxidative stress [170]. Although vitamin E intake reduced carbonyl and MDA levels in a mouse model of COPD, most clinical trials of vitamin supplementation in patients with COPD were ineffective. Vitamin E reduces oxidative stress but has no therapeutic effects on COPD [171].

Plant polyphenols, including flavones, flavanols, and isoflavones, are well-known antioxidants. Resveratrol, a food polyphenol found in red fruits and red wine, is known to have antioxidant effects. It has also been shown to improve GSH synthesis in respiratory epithelial cells by activating the Nrf2 pathway [172]. Patients with COPD can improve their symptoms and lung function by increasing their polyphenol intake. However, a diet that emphasizes fruits and vegetables has failed to control oxidative stress in patients with moderate-to-severe COPD [173], and there is no evidence to prove that it improves COPD. Because resveratrol has low bioavailability, it is desirable to develop a substance with good absorption. Quercetin, a flavanol found in apples, onions, green tea, and capers, also has antioxidant properties and has been shown to prevent the development of emphysema in a mouse model of COPD [174]. Curcumin regulates NF-κB, cyclooxygenase-2 expression, and neutrophil migration in airways and can suppress COPD inflammation by maintaining monocyte histone deacetylase activity, ameliorating impaired steroid responsiveness, and improving the decline in intracellular GSH levels [175].

Research in this area is still ongoing, and it is expected that future studies may reveal pharmaceutical and bioactive compounds that are actually protective for lung health in COPD (Table 3).

## 12. Conclusions

COPD is characterized by emphysema and chronic inflammation due to environmental factors, mainly exposure to smoking, and is associated with acute exacerbations, pneumonia, and lung cancer. Oxidative stress is one of the crucial mechanisms underlying COPD development and is caused by the disruption of the prooxidant–antioxidant balance. Oxidative stress in the lungs is enhanced by ROS released from activated inflammatory cells, such as neutrophils, macrophages, and resident cells, in addition to environmental exposure, such as cigarette smoke, air pollutants, and genetic factors. Furthermore, systemic oxidative stress may be a causal link in COPD comorbidities such as cardiovascular diseases, metabolic syndrome, skeletal muscle wasting, and lung cancer.

Various biomarkers associated with oxidative stress are increased in the blood and exhaled fluid of patients with COPD, and antioxidants that can eliminate oxidative stress are decreased. It is necessary to develop not only standardized biomarkers that can monitor the progression and deterioration of COPD and response to treatment but also effective therapeutic interventions aimed at correcting prooxidant–antioxidant imbalances in COPD.

## Figures and Tables

**Table 1:** Summary of oxidative blood biomarkers in COPD.

Formation of Modified Molecules by Reactive Oxygen Species		COPD vs. Healthy	In COPD Stages
Malondialdehyde		↑[84,85,91,92,108,111,112,113,114,115,116,117] /n.d.[118,119]	↑[85,115,116]/n.d.[114]
8-isoprostane		↑[89]	
Conjugated dienes		↑[113]	
Protein carbonyls		↑[[91,114,120,121] /n.d.[122]	n.d.[114]
Advanced oxidation protein products		↑[92,114]	n.d.[114]
Total hydroperoxides		↑[92,93,123,124]	
Oxidatively damaged DNA		↑[91,114,125] /n.d.[84,120]	
**Antioxidative Molecules and Enzymes**		**COPD vs. Healthy**	**In COPD Stages**
Protein sulfhydryl groups		↓[96,115,118] /n.d.[114,119]	↓[115,126]
Reduced Glutathione	Erythrocyte	↓[85]	↓[85]
	Plasma	↓[108,112,114,116,119] /n.d.[118]	n.d.[114,116]
Biological antioxidative potential		↓[92,114,127,128]	n.d.[114]
Superoxide dismutase activity	Erythrocyte	↑[113]/↓[85,116] /n.d.[114]	↓[116]
	Plasma	↓[92,128] /n.d.[120,123]	
Catalase activity	Erythrocyte	↓[85,116] /n.d.[113,114]	↓[85,116] /n.d.[114]
	Plasma	↓[108,112,128] /n.d.[120]	
Glutathione peroxidase activity	Erythrocyte	↓[85,113,116]	↓[116,127]
	Plasma	↓[108,112] /↑[114]	

↑: increased levels; ↓: reduced levels; n.d.: no significant difference.

**Table 2:** Oxidative stress and genetic factors.

Gene (Status)	Characteristics	Phenotype/Role in COPD
***CYP1A1***	CYP1A1: Production of aromatic hydrocarbon hydroxylase (xenobiotic-metabolizing enzyme)	Gene polymorphisms associated with increased MDA (oxidative stress marker) [112]
***CYP1A2***	CYP1A2: Xenobiotic-metabolizing enzyme, induced by cigarette smoke	
***CYBA***	CYBA: Formation of NADPH oxidase	
***GSTM1***	GSTM1, GSTP1: Detoxification of electrophilic compounds, including products of oxidative stress [156]	Rapid decline in lung function [144]
***GSTP1***		Rapid decline in lung function, FEV_1_ decline, Emphysema [144]
***SOD3***	SOD3: Catalyzes the dismutation of O_2_^−^ into H_2_O_2_ [157]	Rapid decline in lung function [144]
***HDAC2*** **(downregulation)**	HDAC2: Facilitating the formation of transcription repressor complexes [158]	Impairment of Nrf2 activation in the lung [145]
***SIRT1***	SIRT1, 6: Type III HDAC that catalyze NAD^+^-dependent deacetylation	Promotes proteosomal degradation [148]
***SIRT1, SIRT6*** **(downregulation)**		Accelerating ageing of the lung and increased oxidative stress [146,147]
***GSTCD***	GSTCD: Detoxification of products of oxidative stress and synthesis of steroid hormones, lacking key functional domains important for GST activity [150]	Related to FEV_1_ (Positive correlation with mRNA expression) [150,159]
***AGER***	RAGE: multiligand receptor, one of its ligands, AGE, is induced by oxidative stress	FEV_1_ decline [150]
***FAM13A***	FAM13A: Regulating CPT1A expression and fatty acid oxidation [154]	Regulation oxidative stress [143]
***SERPINA1***	Encoding alpha-1-antitrypsin (AAT), inhibiting proteolytic enzymes	A major genetic risk for COPD, contributing to oxidative stress [155]

Abbreviations: CYP, cytochrome p450; CYBA, cytochrome B-245 alpha chain; GST, glutathione S-transferase; MDA, malondialdehyde; GSTM1, glutathione S -transferase Mu 1; GSTP1, glutathione S-transferase pi; SOD3, extracellular superoxide dismutase; O_2_^−^, superoxide anion; H_2_O_2_, hydrogen peroxide; HDAC, histone deacetylase; SIRT1, Sirtuin 1; SIRT6, Sirtuin 6; GSTSD, C-terminal containing domain; CPT1A, carnitine palmitoyl transferase-1A; RAGE, receptor for advanced glycation end-product; AGE, advanced glycation end-product; FEV1, forced expiratory volume in 1 second; FAM13A, family with sequence similarity 13 member A.

**Table 3:** Antioxidant therapeutic interventions in COPD.

Antioxidants	Examples	Studies in COPD
Thiol compounds	*N*-acetylcysteine Carbocysteine Erdosteine	Reduced exacerbation Reduced exacerbation Reduced exacerbation
Nrf2 activators	Sulforaphane Bardoxolone methyl Dimethyl fumarate	Clinical trial negative Effective in animal models Not tested
Plant-derived polyphenols	Resveratrol Quercetin Curcumin	Anti-inflammatory in vitro No clinical trials Anti-inflammatory in vivo
Dietary antioxidants	Vitamin C (ascorbic acid) Vitamin E (α-tocopherol)	No clinical trials No clinical trials

Abbreviations: Nrf2: nuclear erythroid-2 related factor 2.
